# Supplementary figures and images for: Taxonomic reassessment of the genus Dichotomius (Coleoptera: Scarabaeinae) through integrative taxonomy
Source: PeerJ. 2019 Aug 5;7:e7332. doi: 10.7717/peerj.7332 (PMC6686840; doi:10.7717/peerj.7332)

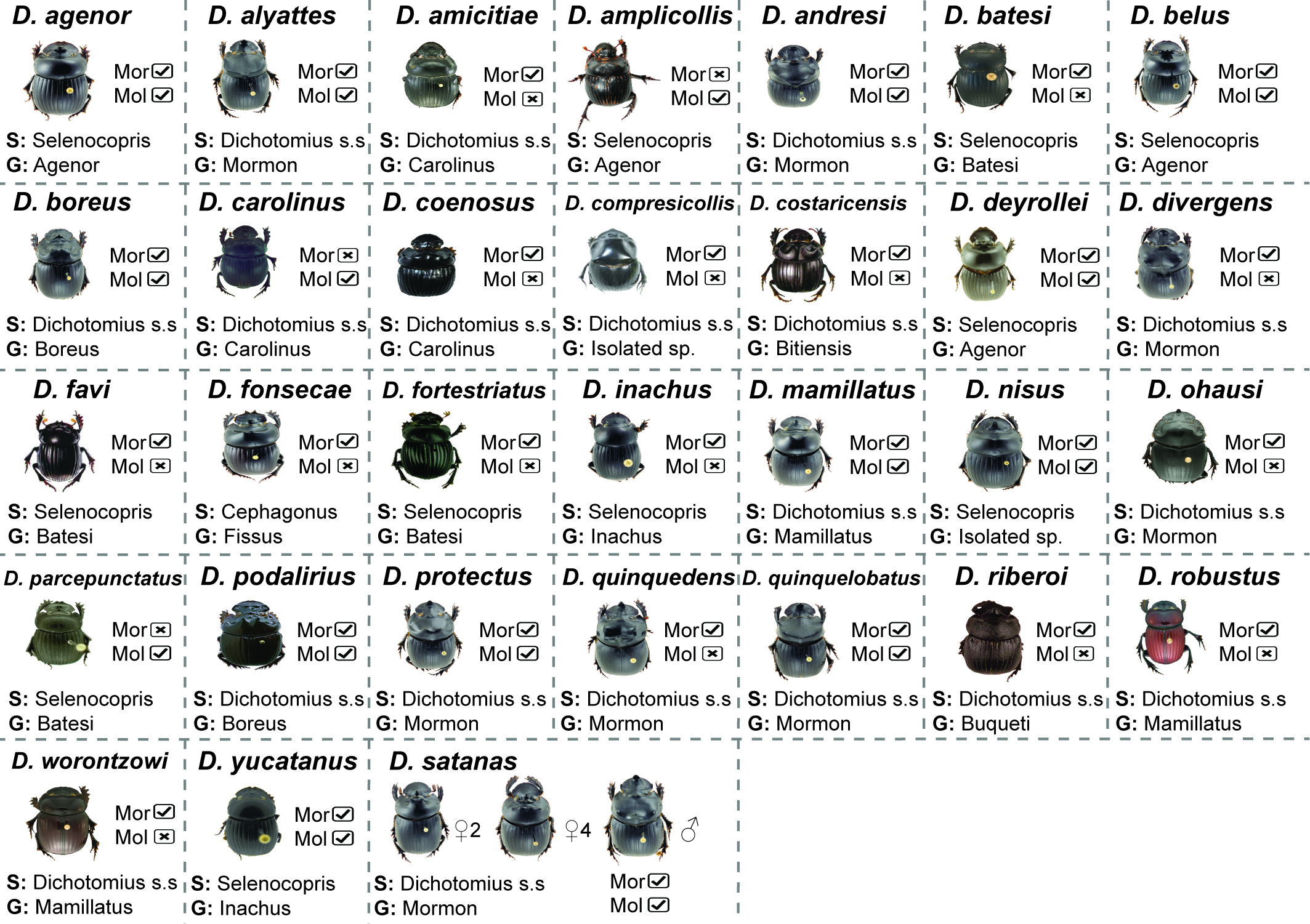

Supplement: Figure S1 — We show subgenus (S) and species-group (G). The type of data available for each species is also indicated as Mor (morphology) and Mol (molecular). For D. satanas we show the female polymorphism based on the number of protuberances in the pronotum, either two (2♂) or four (2♂). Photo credit: Alejandro Lopera. [file peerj-07-7332-s001.jpg]

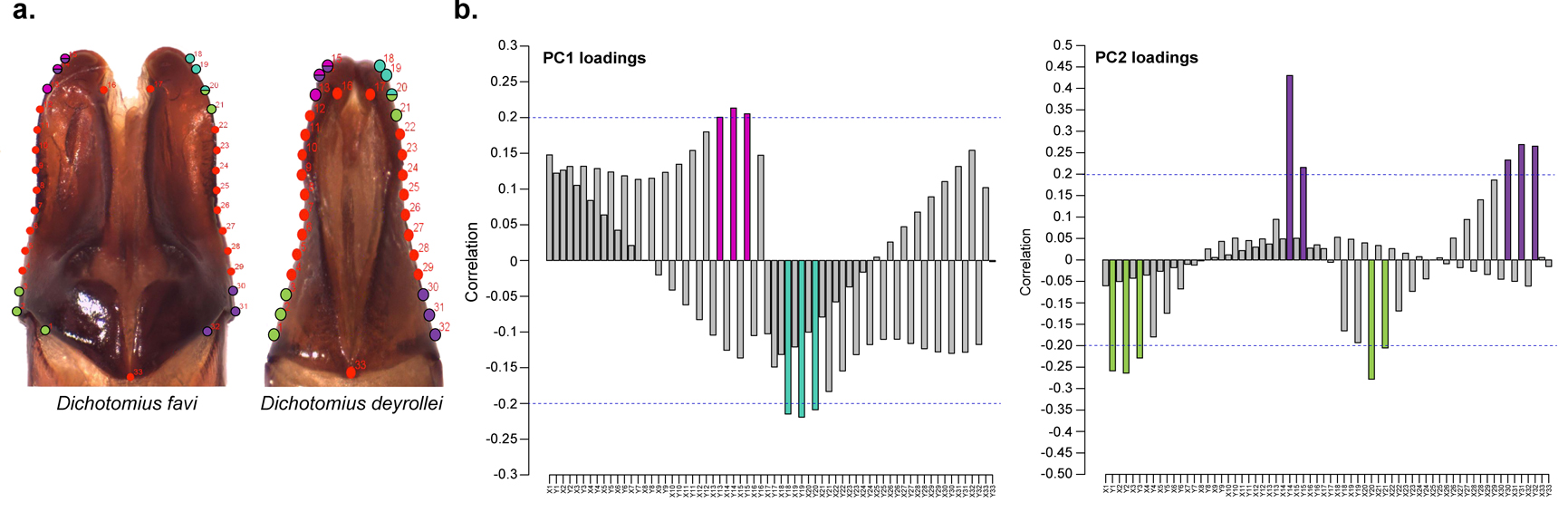

Supplement: Figure S2 — (A) The aedeagus of D. favi and D. deyrollei show the placement of the 33 landmarks used in the geometric morphometric analysis and exemplify extreme differences among species in this trait. (B) PCAloadings of the first two PCs and the correlation of each landmark; landmarks with higher correlation (>0.2) are coloured and their position in aedeagus is highlighted in panel (a) using the same colour. [file peerj-07-7332-s002.jpg]

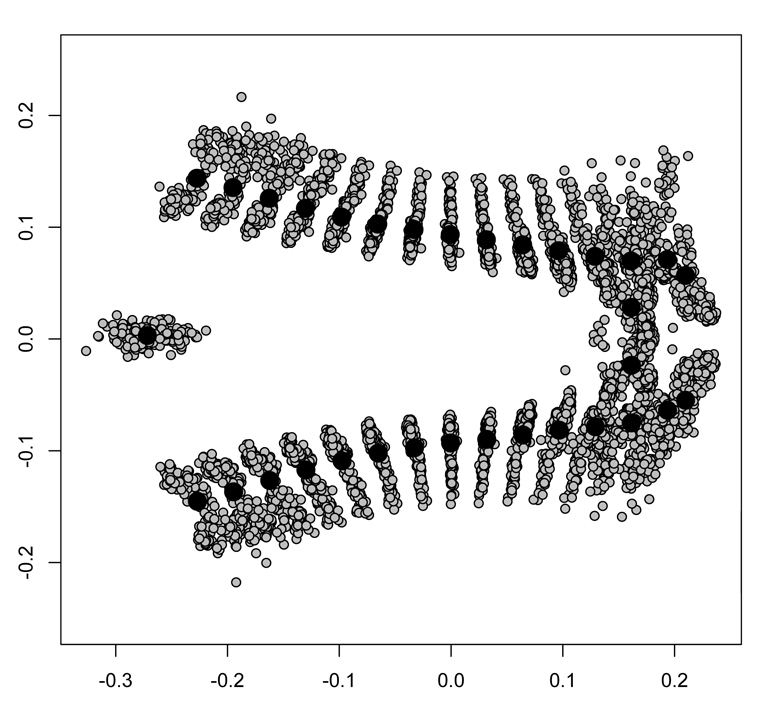

Supplement: Figure S3 — The position of each landmark is also shown. [file peerj-07-7332-s003.jpg]

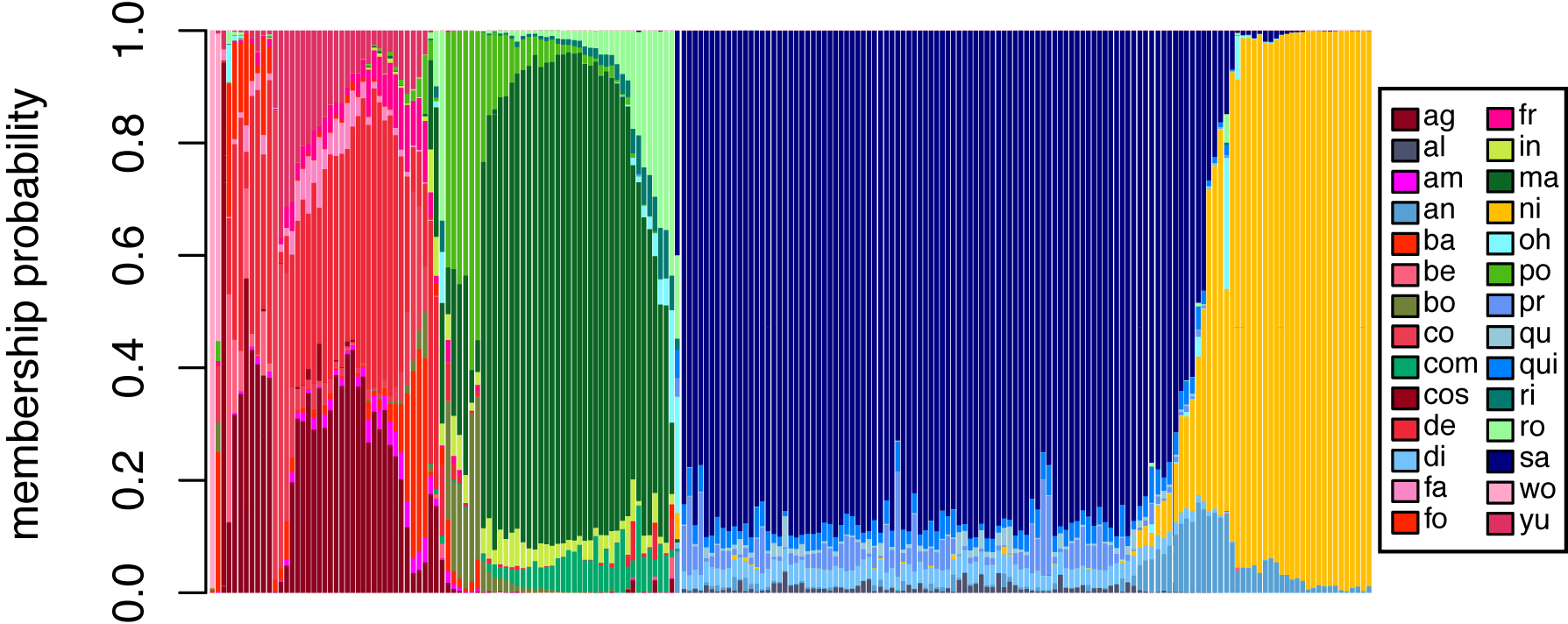

Supplement: Figure S4 — Species identity was used as prior information. Each individual is represented by a vertical bar that is partitioned into coloured segments representing the estimated membership fraction of that individual to a given species. [file peerj-07-7332-s004.jpg]

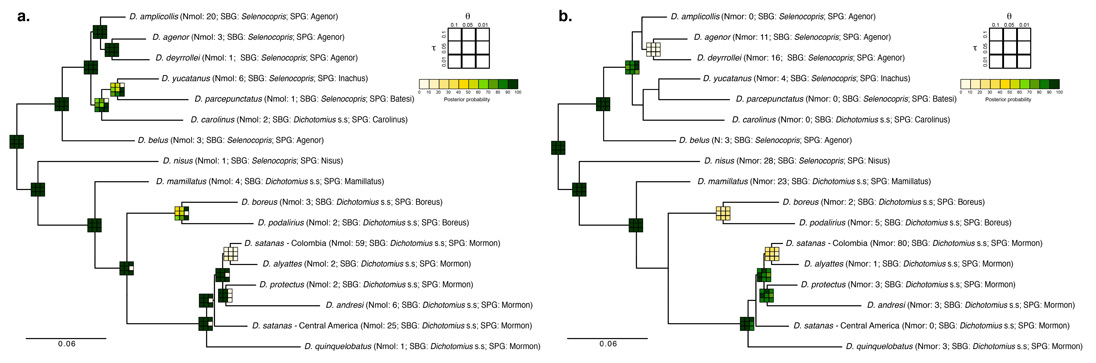

Supplement: Figure S6 — Mean posterior probabilities of Bayesian species delimitations were inferred under 9 different theta and tau prior combinations. The posterior probability of each of these combinations is colour-coded and indicated in 3 × 3 boxes on each node of the guide tree. Nodes with no box in panel (B) are those where at least one species lacked data. The large 3 × 3 inset indicate the position of each prior combination in these boxes. Next to the species name we indicate the number of individuals included per species in the analysis (Nmol: number of individuals with molecular data in (A) and Nmor: number of individuals with morphological data in (B)). Subgenus (SBG) and species group (SPG) are also indicated. [file peerj-07-7332-s006.jpg]

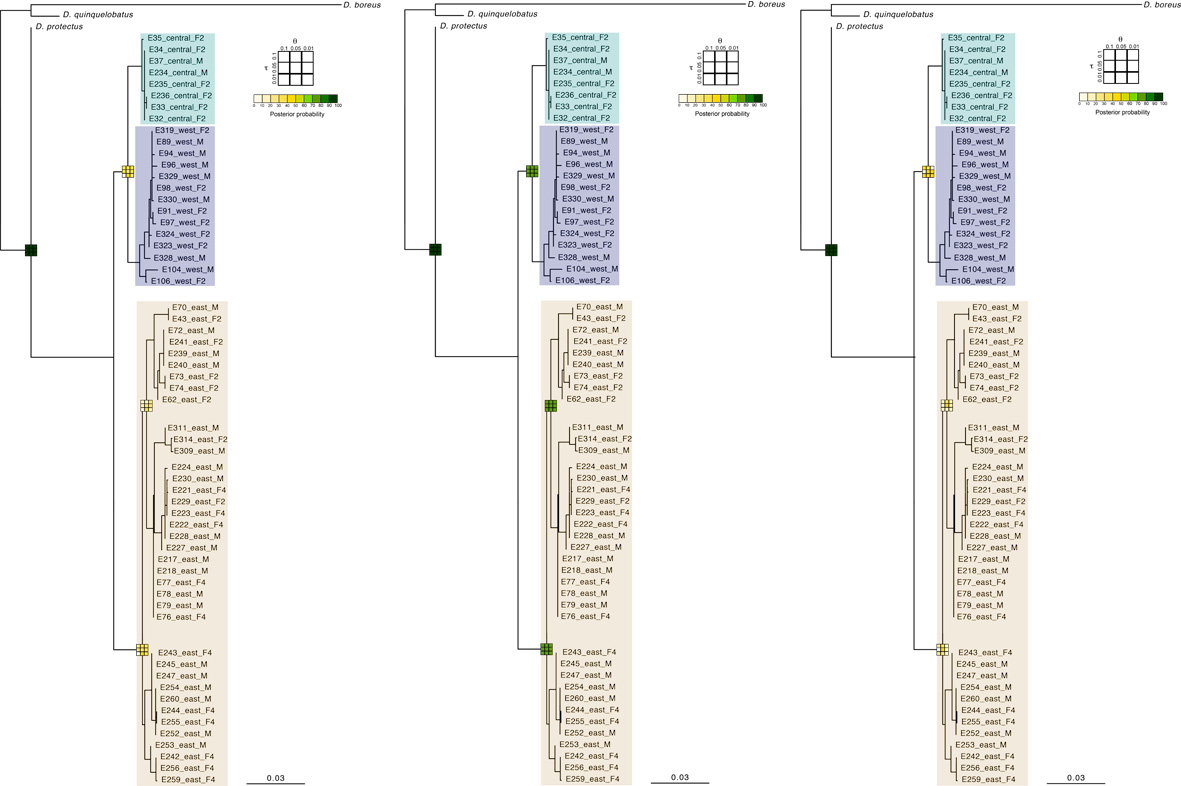

Supplement: Figure S7 — (left) total-evidence, (middle) molecular data alone and (right) morphology data alone. Mean posterior probabilities of Bayesian species delimitations were inferred under 9 different theta and tau prior combinations. The posterior probability of each of these combinations is colour-coded and indicated in 3 × 3 boxes on each node of the guide tree. Nodes with no box in panel (B) are those where at least one species lacked data. The large 3 × 3 inset indicate the position of each prior combination in these boxes. Next to the species name we indicate the number of individuals included per species in the analysis (Nmol, number of individuals with molecular data in (A) and Nmor, number of individuals with morphological data in (B)). Subgenus (SBG) and species group (SPG) are also indicated. [file peerj-07-7332-s007.jpg]
